# Supplementary material for: LDHA Suppression Altering Metabolism Inhibits Tumor Progress by an Organic Arsenical
Source: Int J Mol Sci. 2019 Dec 11;20(24):6239. doi: 10.3390/ijms20246239 (PMC6940739; doi:10.3390/ijms20246239)
Supplement: Supplementary file 1 [file ijms-20-06239-s001.pdf]

Supporting information

A: PDT-PAO

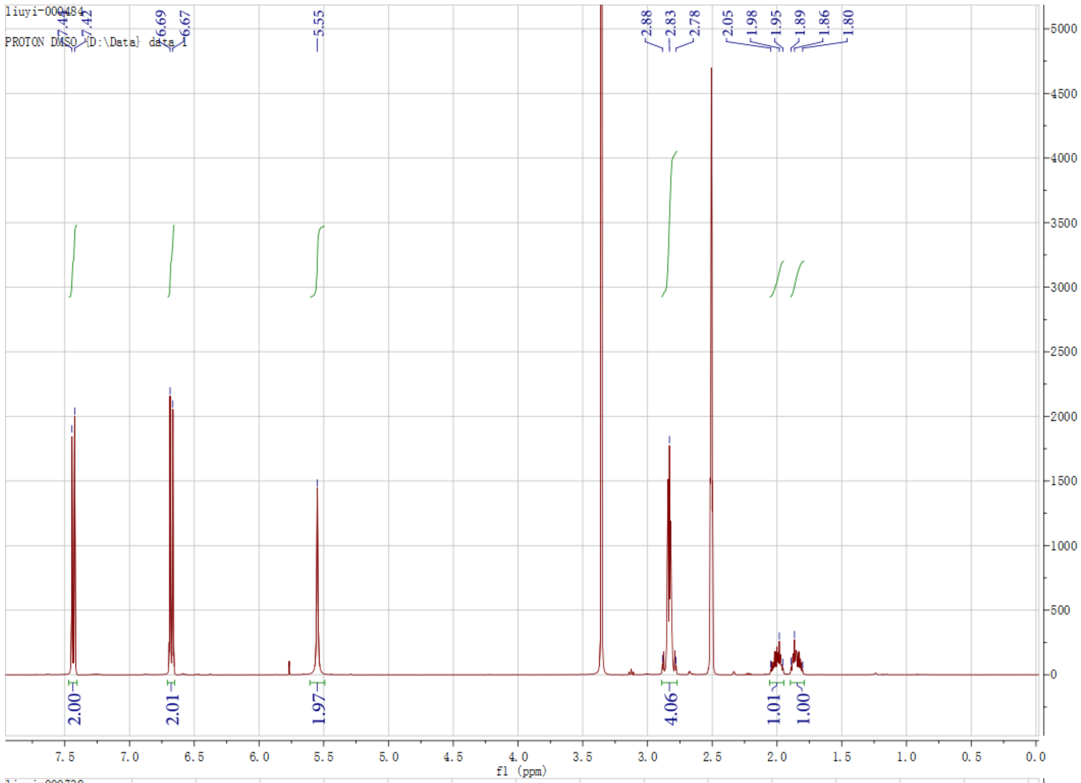

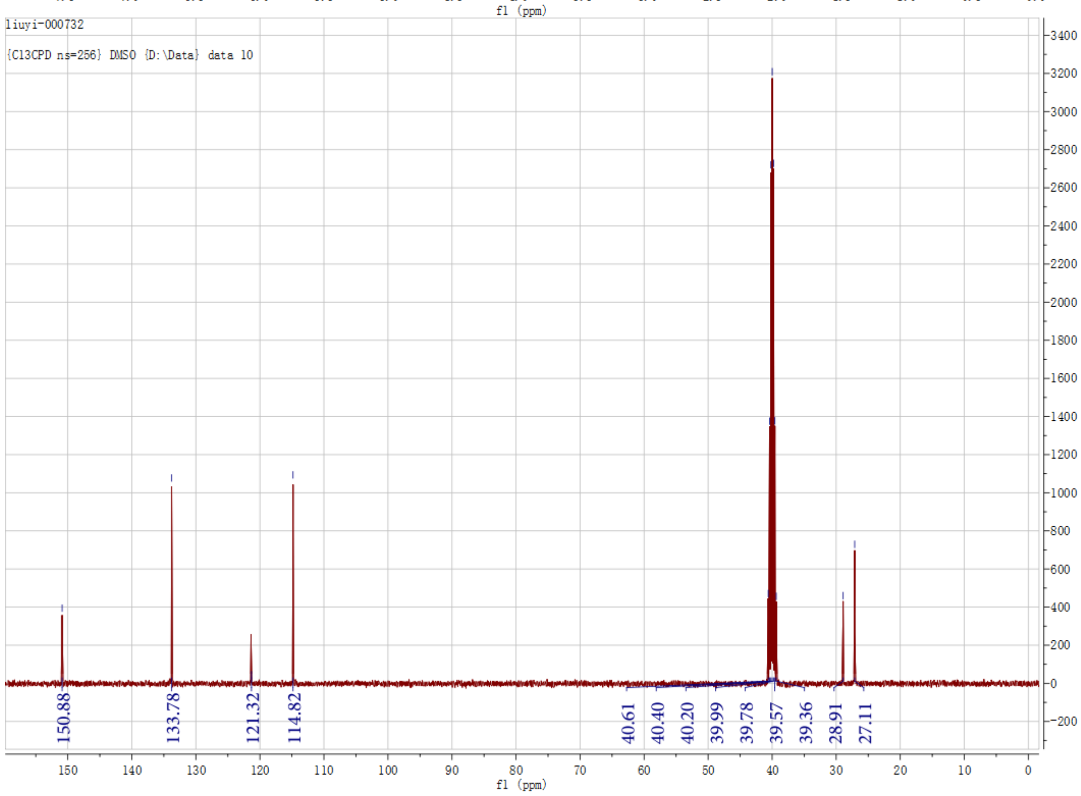

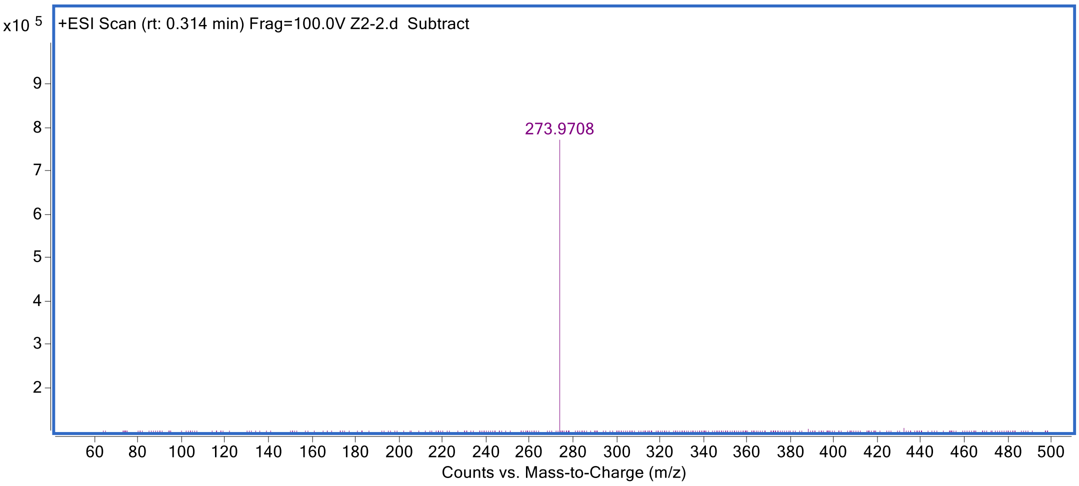
x10 5
+ESI Scan (rt: 0.314 min) Frag=100.0V Z2-2.d Subtract
273.9708
Counts vs. Mass-to-Charge (m/z)

B: PDT-BIPA

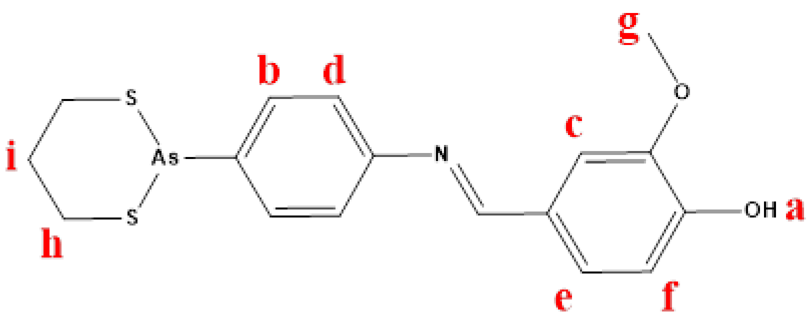
g
O
b
d
c
i
As
N
OH
a
S
S
h
e
f

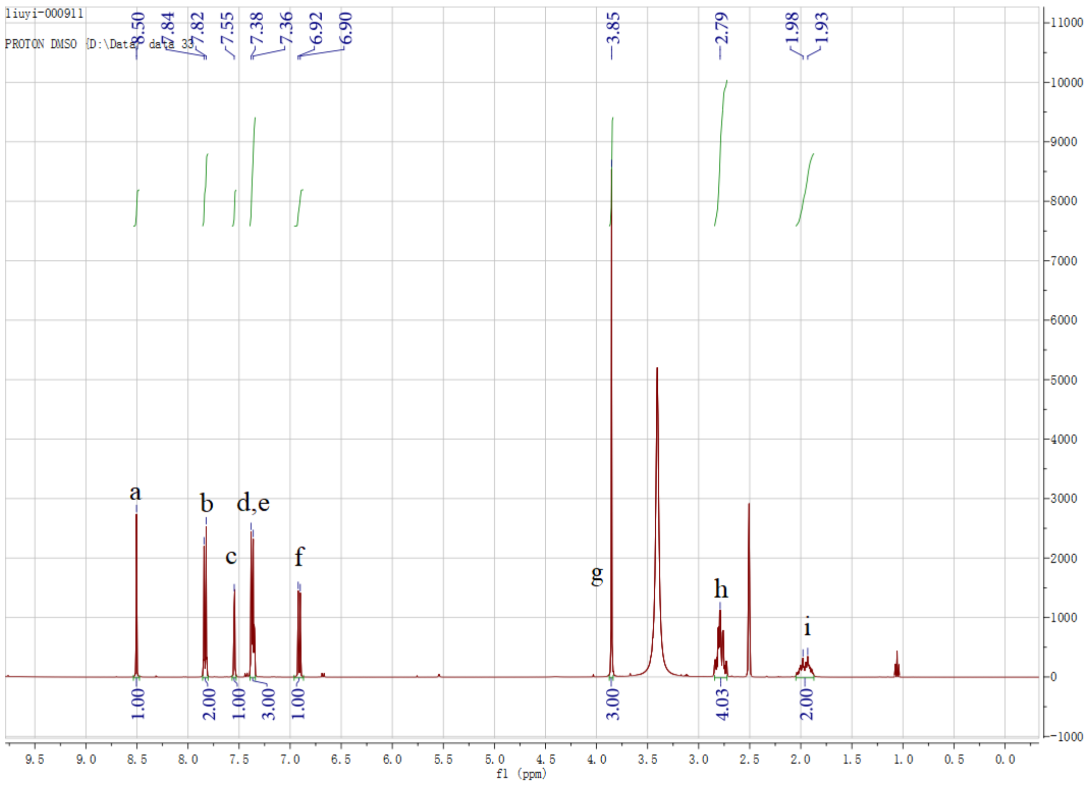
liuyi-000911
PROTON DMSO {D:\Data} data 33
8.50
7.84
7.82
7.55
7.38
7.36
6.92
6.90
3.85
2.79
1.98
1.93
a
b
c
d,e
f
g
h
i
1.00
2.00
1.00
3.00
1.00
3.00
4.03
2.00
f1 (ppm)

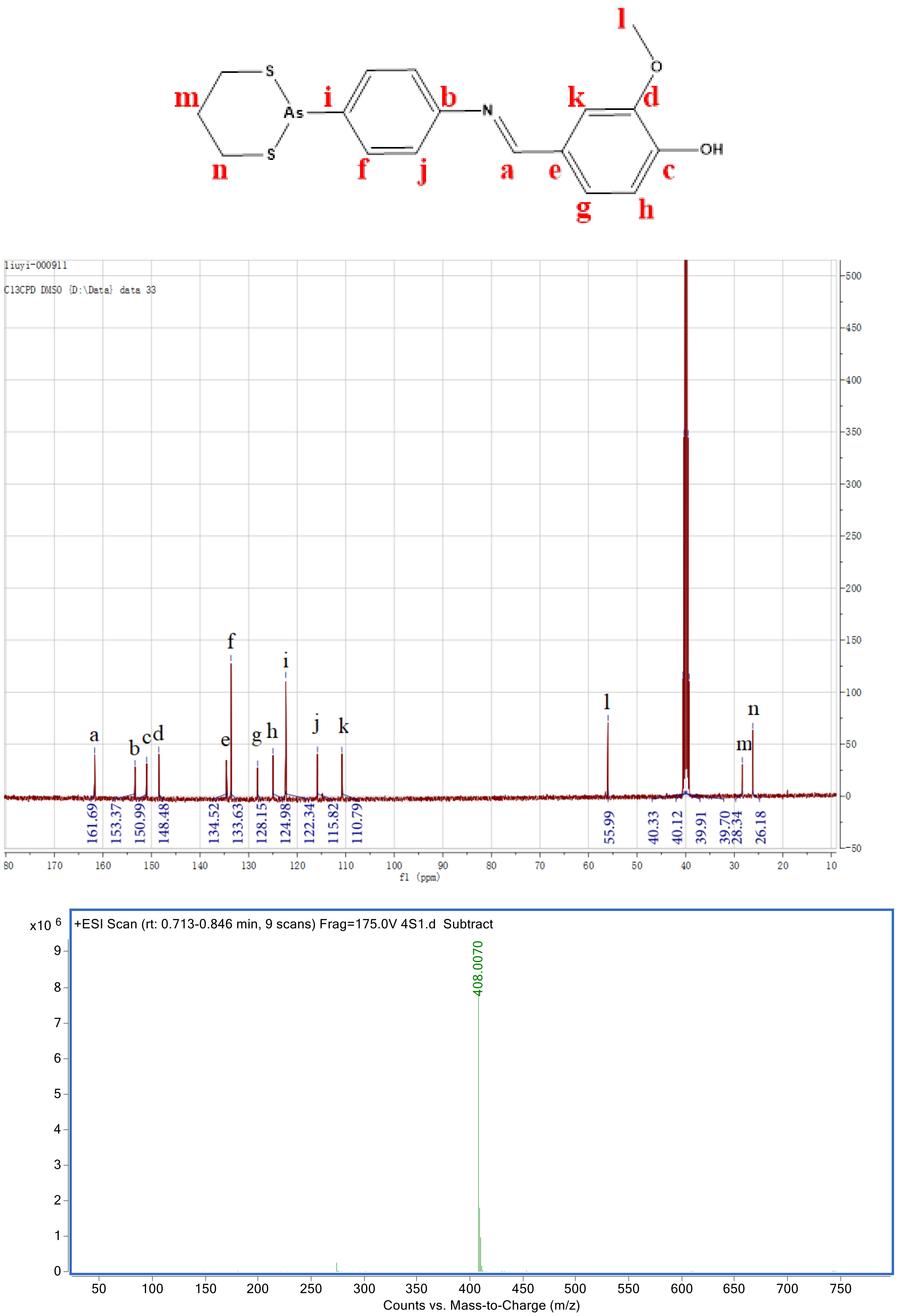

Figure S1. ^{1}H and ^{13}C NMR and Mass spectra of PDT-PAO (A) and PDT-BIPA (B).

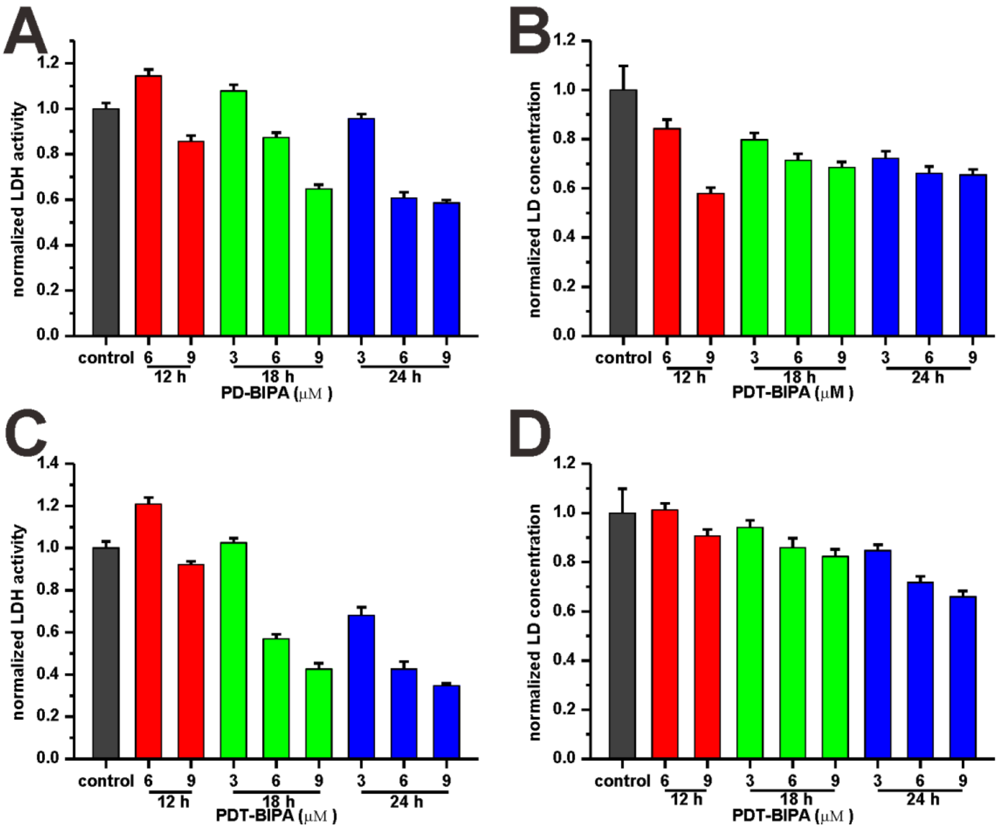

Figure S2. LDH inhibition and LD reduction. GES cells were incubated with PDT-BIPA of several concentrations for 3, 6, 9 h or 12, 18, 24 h. The LDH activity and LD concentration of the cell lysate (A, B) or the medium (C, D) were tested according to the kit instruction.

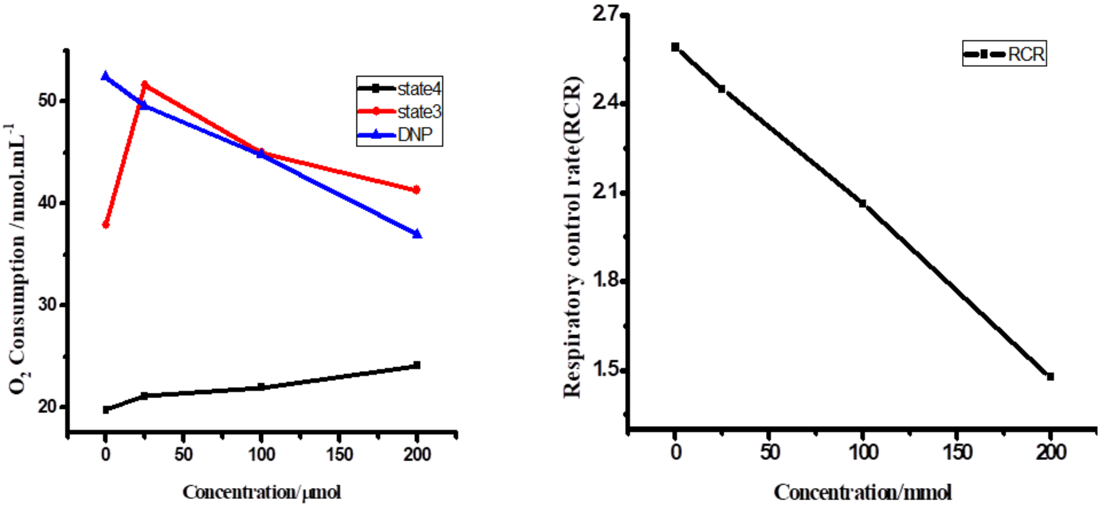

Figure S3. The OCR of isolated mitochondria and the reduced RCR.

Table S1. Influence of PDT-BIPA on mitochondrial metabolism parameters.[a]

As/μM	0	50	100	200

**Q/J**	11.87	11.51	10.17	10.07
**k_1/h^{-1}**	0.2000	0.1809	0.1657	0.1361
**R_1^2**	0.9900	0.9971	0.9967	0.9912
**t_1/h**	30.78	42.51	45.15	52.55
**$P_1/\mu W$**	171.51	168.66	170.99	140.11
**K_2/h^{-1}**	0.0927	0.0731	0.0600	0.1521
**R_2^2**	0.9935	0.9929	0.9907	0.9933
**t_2/h**	36.53	47.31	49.43	55.58
**$P_2/\mu W$**	266.56	232.97	215.78	200.43
**K_3/h^{-1}**	-0.348	-0.274	-0.1923	-0.1253
**R_3^2**	0.9830	0.9849	0.9869	0.9858

[a] k_1: rate constant in activity recovery phase; k_2: rate constant in stationary increase phase; k_3: rate constant in decline phase; R: correlation coefficient; P_m: maximum power output; t_m: maximum power output time; Q: total heat output.
